# Quality of Life, Satisfaction, Occlusal Force, and Halitosis after Direct and Indirect Relining of Inferior Complete Dentures

**DOI:** 10.1055/s-0041-1731838

**Published:** 2021-08-24

**Authors:** Mariana Vilela Sônego, Clóvis Lamartinede Moraes Melo Neto, Daniela Micheline dos Santos, André Luiz de Melo Moreno, André Pinheiro de Magalhães Bertoz, Marcelo Coelho Goiato

**Affiliations:** 1 Department of Dental Materials and Prosthodontics, School of Dentistry, São Paulo State University, Araçatuba, São Paulo, Brazil; 2 Oral Oncology Center, School of Dentistry, São Paulo State University, São Paulo, Brazil; 3 Department of Pediatric and Social Dentistry, School of Dentistry, São Paulo State University, Araçatuba, São Paulo, Brazil

**Keywords:** denture relining, complete denture, quality of life, bite force, dental prosthesis, edentulous mouth, edentulous jaws

## Abstract

**Objective** The aim of this study was to compare the direct relining technique with the indirect relining technique in relation to quality of life, satisfaction with the relining, occlusal force, and halitosis of users of acrylic complete dentures.

**Materials and Methods** Twenty bimaxillary edentulous individuals were selected. They had to use the same pair of complete dentures for a minimum of 1 year and a maximum of 5 years. The subjects were randomly divided in groups of direct relining and indirect relining of the inferior denture ( *n* = 10). Both groups received the same silicone-based relining. The clinical tests verified the quality of life (Oral Health Impact Profile in edentulous individuals), the satisfaction with the relining, the occlusal force, and halitosis. The tests (halitosis and occlusal force) were performed initially (before the relining), immediately after the relining, and 30, 60, 90, and 180 days after the relining. The questionnaires (quality of life and satisfaction with the relining) were performed initially (before the relining), and 30, 60, 90, and 180 days after the relining.

**Statistical Analysis** Analysis of variance and the Tukey test were used ( *p* < 0.05).

**Results** There was no statistical difference comparing the two techniques in all the evaluations ( *p* < 0.05). There was a significant statistical difference for the factor of time in all clinical tests for each relining technique ( *p* < 0.05). The quality of life and satisfaction with the relining increased significantly 30 days after the relining when compared with the initial time point, for both techniques ( *p* < 0.05). The occlusal force increased significantly after 90 and 180 days when compared with the initial time point, for both techniques ( *p* < 0.05). Halitosis decreased significantly immediately after the relining when compared with the initial time point, for both techniques ( *p* < 0.05).

**Conclusion** Independent of the relining technique used, there was an increase in the quality of life, satisfaction with the relining, and occlusal force, as well as a reduction in the level of halitosis. Both techniques generated similar results and therefore can be options in clinical practice.

## Introduction

In 2015, 8.5% of the 7.3 billion people in the world were aged 65 years or older. In 2050, those aged 65 or older will represent ~16.7% of the total world population, estimated to be 9.4 billion people. 1 Therefore, the average annual increase in people aged 65 years or older between 2015 and 2050 will be 27.1 million. 1 Edentulism is a worldwide health problem 2 and can be associated with people aged above 50 years. 1 3 4 Thus, due to the increase in the elderly population that will occur, edentulism can continue to be a worldwide health problem in the future.

Despite the popularity of implant-supported prostheses, questions of health, finance, and/or psychology could impede their use. 2 Thus, an edentulous patient would need to use conventional complete dentures for aesthetic and functional reasons. 2 Therefore, conventional complete dentures can be considered an extremely important treatment option for odontology.

The majority of patients who use complete dentures encounter difficulties in the use of a mandibular denture, due to the fault of retention and stability. 5 6 7 8 This occurs through the continuous process of resorption of the mandibular bone ridge, which can be four times greater in comparison to the maxillary ridge. 8 These functional limitations can damage quality of life, 5 7 9 occlusal force, and the coordination of muscular movements of the individual. 6 10

Relining with a soft material is used to reduce the sensation of discomfort and localized pressure, as well as improving mastication, the distribution of masticatory forces, and the retention and stability of the complete denture. 2 5 7 10 In addition, this procedure avoids lesions of the mucosa and the accelerated resorption of the bone ridge, which can occur due to a maladjustment of a complete denture on the ridge. 11 It is worth emphasizing that the low muscular exertion of patients who use complete dentures, primarily due to advanced age and discomfort of the dentures, could lead to muscular atrophy and the weakening of the mandibular muscles. 2 Thus, the relining of complete dentures is an extremely important treatment for edentulous patients.

Beside the question of function, relining of a complete denture provides a new contact surface between the denture and the mucosa. This is important since complete dentures can accumulate and retain a microbial film over time, 12 13 worsening oral hygiene and affecting the health of patients. 14 15 This situation occurs principally because of the surface roughness of the acrylic resin, which tends to increase through time due to its low resistance to abrasion and daily brushing with dental pastes containing abrasives. 14 15 16 It is important to emphasize that the accumulation of microbial plaque on the complete denture can lead to bad breath. 12 13

The relining of a complete denture can be done in a direct manner in the clinic, or indirectly, combining the clinical and laboratorial phase. 11 16 There are no known studies in the literature that compare these techniques clinically with a silicone-based reliner. Therefore, the objective of the present study was to compare the direct relining technique with the indirect relining technique in relation to quality of life, satisfaction with the relining, occlusal force, and halitosis of complete dentures users.

## Materials and Methods

This study was approved by the Human Research Ethics Committee of the São Paulo State University (School of Dentistry, Araçatuba, São Paulo, Brazil) with the number 48606215500005420. All patients gave their informed consent prior to their inclusion in the study.

Twenty individuals drawn from UNESP (São Paulo State University, School of Dentistry, Araçatuba, São Paulo, Brazil) were selected. These individuals were randomly divided in two groups ( *n* = 10). Group 1 received relining by using the direct method. Group 2 received relining by using the indirect method. The relining was performed only on the inferior denture of each individual, 2 5 10 due to its instability. 2 6 7 8 The individuals in each group did not know the difference between the treatments performed.

### Inclusion Criteria

- This study included users of bimaxillary acrylic complete dentures from 40 to 80 years old, 2 5 with complete dentures without fractures and with unworn cuspids, 2 5 17 with an unstable inferior denture, without retention, and which could cause pain. 7 9
- Individuals with a favorable maxillary-mandibular relation 2 5 18 and with a correct occlusal vertical dimension 2 5 based on aesthetic techniques, physiology, 9 and pleasure. 19
- Individuals classified by the American Society of Anesthesiologists (ASA) as ASA 1 or ASA 2 (controlled systemic disease), 20 with cognitive ability to respond to questions 9 ; which used the same pair of complete dentures for a minimum of 1 year and a maximum of 5 years, 7 with moderate resorption of the mandibular alveolar ridge 2 5 10 which did not use fixation adhesives for complete dentures.
- The maxillary ridge must provide retention to the upper denture.

### Exclusion Criteria

- This study excluded individuals who were carriers of oral pathologies in soft or hard tissues, 2 5 with implants or residual roots, 2 5 or who were incapable of responding to the questions in the questionnaires for any reason. 5
- Those who drink alcohol frequently.
- Traumatic injuries in oral tissues.
- Maxillary and / or mandibular torus.
- Individuals with neurological illnesses or without motor coordinations. 2 5
- Temporomandibular dysfunction, verified by the Research Diagnostic Criteria. 9 17
- Individuals with tumors. 17
- Patients undergoing radiotherapy or chemotherapy treatment.
- Smokers.
- Use of illicit drugs.
- Individuals with partial or total dependency of care by third parties.

### Time Points at Which Tests and Questionnaires Were Performed

The clinical interventions were performed by one single professional. The halitosis and occlusal force tests and questionnaires (quality of life and satisfaction with the relining) were applied by one single examiner, who was not the professional that performed the clinical interventions on the patients, and in a manner not knowing what group each patient belonged to. The laboratory phase was always performed in the same prosthesis laboratory and by the same prosthetic technician.

The tests (halitosis and occlusal force) were performed initially (before the relining), immediately after the relining, and 30, 60, 90, and 180 days after the relining. The time points “initial” and “immediately after the relining” were on different days. The questionnaires (quality of life and satisfaction with the relining) were performed initially (before the relining) and 30, 60, 90, and 180 days after the relining. The time point “immediately after the relining” was not included in the evaluations using the questionnaires, as patients needed to use their dentures for a period of time.

### Occlusal Force

A digital dynamometer (Model IDDK, Kratos, Brazil) was used. 6 The evaluations were done in the regions of the first molar (right and left) and in the region of the central incisors. Each individual bit the device three times in each region with maximum force. The rest between each recording was from 2 to 3 minutes. 6 The maximum occlusal force was recorded in kgf. After performing the test three times in each region, the highest value in each region was recorded. 6

### Halitosis Test

A Halimeter (Breath Alert, TANITA, Japan) was used for the measurement of halitosis. 21 22 23 This device quantifies the levels of volatile sulfur compounds that cause bad oral odor. 21 22 The values of this device vary from 1 to 4, being: 1—absence of odor (normal); 2—light odor (normal); 3—moderate odor (bad breath—perceptible); and 4—strong odor (bad breath—perceptible). 21 22 23 The assessments were performed according to the orientation of the manufacturer, leaving the sensor a distance of ~1 cm from the half-open mouth. After examining each patient, the air opening was cleaned with a dry cloth and the Halimeter was gently shaken four to five times in the air to remove any odors or moisture left in product. 22 23

The halitosis test was performed at each time point (initial, immediately after relining, and after 30, 60, 90, and 180 days after relining) in the following sequence:

1. Evaluation of halitosis with dentures and without dentures—patients were evaluated as soon as they arrived at the dental clinic; the only exception was on the days when the relining techniques were performed, because the patients’ halitosis was measured after the techniques were performed (“immediately after relining”). The measurements of halitosis were performed first with the dentures and then without the dentures.
2. Evaluation of halitosis with dentures and without dentures immediately after cleaning the mouth and dentures. No type of hygiene guidance was recommended to the research participants. Each patient performed their hygiene, based on their own knowledge. Patients were asked to take the products they used to perform their oral hygiene to the clinic on the days of the evaluation. The measurements of halitosis were performed first with the dentures and then without the dentures.

The halitosis test was performed three times at each time point to confirm the results. Therefore, for each patient, the value of the Halimeter (1, 2, 3, or 4) that was repeated at least 2 times was recorded. If no number appeared, it was considered a reading error and the procedure was repeated. 22

In this study, mean values of halitosis level < 3 were considered clinically acceptable, and mean values ≥ 3 were considered clinically unacceptable.

### Relining Procedure According to the Manufacturer

The material used in the clinic for all of the relining was UFI Gel SC (VOCO, Germany). This product is a polyvinyl siloxane-based reliner of long duration (up to 2 years).

Initially, the dentures were cleaned with a toothbrush and subsequently immersed in denture cleaning solution (Corega Tabs, GSK, Brazil), to remove residues on their bases and acrylic teeth. These procedures were important to avoid failure of adhesion of the reliner to the base of the denture. Gauze was passed over the edges of the maxilla and mandible to remove debris. Finally, the patient was asked to perform a mouthwash with water.

The occlusal vertical dimension of the patient was measured with a compass, which measured the distance between two previous marked points, being on the nose and the on the chin. 19 Then, a 2 mm wear of the inferior complete denture base was performed with a Maxicut 1251 tungsten bit (Edenta, Switzerland), followed by the rounding of its margins.

For Group 1, after wear, the inferior complete denture was cleaned with cotton embedded with 90% alcohol, and after 1 minute of drying, the adhesive (VOCO) was applied. One minute after the application of the adhesive (VOCO), the reliner was applied over the inferior complete denture base, and after 1 more minute the inferior denture was carried to the mouth of the patient. The patient was instructed to occlude their acrylic teeth until the occlusal vertical dimension was reestablished, that is, at the moment in which the compass united the two marked points. Afterward, the occlusion was maintained for 1 minute. Next, it was recommended that the patient do movements of mastication and swallowing for 5 minutes. After 6 minutes in total, the inferior complete denture was removed from the mouth. The finishing of the inferior denture was performed with a scalpel blade, scissors, and polishing discs (VOCO, Germany) 30 minutes after its removal from the mouth of the patient. Then, the glazing was applied over the relining to seal and smooth this surface, following the manufacturer's recommendations.

For Group 2, after applying a universal adhesive (Universal Tray Adhesive, Zhermack, Italy) over the base of the worn inferior denture (2 mm), silicone (light-addition silicone, Express XT, 3M ESPE, United States) was placed over the denture base, and it was positioned on the mandibular ridge. The patient was asked to perform movements of the tongue. Then the patient was asked to close until the occlusal vertical dimension was reestablished, with the same principle as mentioned earlier (if necessary, the mold was refilled). After molding and disinfecting the mold (hypochlorite), 24 type IV plaster (Durone, Dentsply, Brazil) was dispensed over the mold to obtain the inferior plaster model. The other procedures were in accordance with the manufacturer's recommendations. The finishing was performed in the same manner for Group 1. While the laboratory procedures were being performed, the patient waited at the clinic. Before placing the inferior denture in the patient's mouth, it was disinfected with hypochlorite. 24

The participants returned for possible adjustments after 24, 48, and 72 hours. 5

### Oral Health Impact Profile in Edentulous Individuals and Satisfaction Questionnaire with the Relining

The Oral Health Impact Profile in edentulous individuals (OHIP-EDENT—Brazil) is composed of 19 questions evaluating handicap, social disability, psychological disability, physical disability, psychological discomfort, physical pain, and functional limitation. ^25^ Thus, this questionnaire assesses oral health-related quality of life. For each question, the patient could choose one of the following answers: “never”; “sometimes”; or “always”. 25

A questionnaire with nine specific questions was developed to verify satisfaction with the relining. For each question, the patient could choose one of the following answers: “never”; “sometimes”; or “always”. The questions were:

1. Do you have some painful sensation in the mouth?
2. Do you taste some unpleasant taste in your mouth?
3. Do you smell some odor coming from the complete dentures?
4. Do you perceive that there is retention of food in the inferior complete dentures?
5. Do you feel your complete dentures are unstable?
6. Do you feel difficulty cleaning the complete dentures?
7. Do you feel that the inferior complete denture base is rough?
8. Do you notice a loss of reliner material adhered to the complete denture?
9. Do you feel that the relining material is hard?

Scores from 1 to 3 for each question were assigned to these two questionnaires according to the answers of the patients, being “1” corresponding to “never,” “2” equal to “sometimes,” and “3” corresponding to “always.” The sum of these values in each questionnaire corresponded to quality of life and satisfaction with the relining. Thus, the smaller the sum of the values in each questionnaire, the greater the quality of life and satisfaction with the relining.

### Statistical Analysis

Statistical analysis of the results was performed by using a statistical software program (IBM SPSS Statistics, v24.0, IBM Corp, United States). The statistical analysis was performed by the analysis of variance and the Tukey test. The level of significance was 5%.

## Results

In this study, 75% of the patients were female. The mean age of the participants was 67.3 years and the mean time of use of the same pair of complete dentures was 4.5 years. All cleaned their complete dentures with a brush and dental paste. For the hygiene of oral tissue, the most common method was the use of a brush and toothpaste ( *n* = 13), followed by mouthwash with water ( *n* = 5) and lastly, mouthwash with Listerine (Johnson & Johnson, Brazil) ( *n* = 2).

### Quality of Life

For quality of life, there was no difference between the techniques ( *p* = 0.608). There was a significant statistical difference between the time points evaluated for each technique ( *p* < 0.001). After 30 days of relining, quality of life increased significantly when compared with the initial time point, for both techniques ( *p* < 0.05). There was no significant difference in quality of life between time points 30, 60, 90, and 180 days after relining, for both techniques ( *p >* 0.05; Table 1 ).

**Table 1:** Mean and standard deviation of quality of life for each time point and relining technique

Time points	Techniques	
	Direct	Indirect
Initial	35.6 (6.6) Aa	34.1 (4.7) Aa
30 d	24.1 (2.4) Ba	23.7 (2.7) Ba
60 d	24.3 (2.9) Ba	25.0 (2.8) Ba
90 d	24.2 (3.1) Ba	24.7 (2.5) Ba
180 d	23.2 (1.9) Ba	23.1 (2.3) Ba
Note: Tukey test ( *p* < 0.05). Different capital letters in vertical (column) show a statistically significant difference ( *p* < 0.05). Different lower case letters in horizontal (line) show a statistically significant difference ( *p* < 0.05).		

### Satisfaction with the Relining

There was no difference between the relining techniques ( *p* =0.170). There was a difference between the time points for each technique ( *p* < 0.01). After 30 days of relining, satisfaction with the relining increased significantly when compared with the initial time point, for both techniques ( *p* <0.05). There was no significant difference in satisfaction between time points 30, 60, and 90 days after relining, for both techniques ( *p >* 0.05). After 180 days of relining, there was a reduction in satisfaction when compared with time points 30, 60, and 90 days, for both techniques ( *p* < 0.05; Table 2 ).

**Table 2:** Mean and standard deviation of satisfaction for each time point and relining technique

Time points	Techniques	
	Direct	Indirect
Initial	13.0 (3.3) Aa	13.9 (1.9) Aa
30 d	10.1 (0.8) Ba	9.7 (1.2) Ba
60 d	10.0 (0.9) Ba	10.5 (1.0) Ba
90 d	11.6 (1.0) Ba	11.7 (2.0) Ba
180 d	13.2 (1.5) Aa	12.4 (1.8) Aa
Note: Tukey test ( *p* < 0.05). Different capital letters in vertical (column) show a statistically significant difference ( *p* < 0.05). Different lower case letters in horizontal (line) show a statistically significant difference ( *p* < 0.05).		

### Occlusal Force

There was no statistical difference between the relining techniques ( *p* < 0.05). There was a difference between the time points evaluated for each technique ( *p* < 0.01). The occlusal force increased significantly after 90 and 180 days when compared with the initial time point, for both techniques ( *p* <0.05), and for the three regions evaluated ( *p* < 0.05; Table 3 ).

**Table 3:** Mean (kgf) and standard deviation of occlusal force for each time point and relining technique

Region	Time points	Techniques	
		Direct	Indirect
Right molar	Initial	3.92 (1.3) Aa	4.50 (1.6) Aa
	Immediately after the relining	4.00 (2.1) Aa	3.54 (1.5) Aa
	30 d	5.25 (2.1) ABa	5.15 (1.7) ABa
	60 d	4.88 (1.6) ABa	5.27 (1.9) ABa
	90 d	6.59 (1.5) BCa	6.49 (1.6) BCa
	180 d	8.00 (2.2) Ca	6.47 (1.9) Ca
Central incisors	Initial	2.82 (1.1) Aa	2.96 (1.3) Aa
	Immediately after relining	2.52 (1.3) Aa	2.51 (1.1) Aa
	30 d	3.85 (1.6) Aa	3.60 (1.3) Aa
	60 d	3.34 (1.7) Aa	4.20 (1.2) Aa
	90 d	5.43 (1.1) Ba	5.03 (1.4) Ba
	180 d	6.43 (2.0) Ba	4.78 (1.6) Ba
Left molar	Initial	4.16 (2.0) Aa	4.78 (1.8) Aa
	Immediately after relining	4.22 (2.6) Aa	3.27 (1.3) Aa
	30 d	5.00 (2.1) ABa	5.18 (2.4) ABa
	60 d	5.36 (1.8) ABCa	5.51 (2.2) ABCa
	90 d	6.76 (1.3) BCa	6.52 (1.8) BCa
	180 d	8.03 (1.9) Ca	6.33 (1.4) Ca
Note: Tukey test ( *p* < 0.05). Different capital letters in vertical (column) show a statistically significant difference (in each region individually) ( *p* < 0.05). Different lower case letters in horizontal (line) show a statistically significant difference ( *p* < 0.05).			

### Halitosis

There was no statistical difference between the relining techniques ( *p* < 0.05). There was a difference between the time points evaluated for each technique ( *p* = 0.01). There was a significant reduction in halitosis immediately after relining when compared with the initial time point, in all cases shown in Table 4 , for both techniques ( *p* < 0.05).

**Table 4:** Mean and standard deviation of halitosis for each time point and relining technique

Presence of dentures and hygiene	Time points	Techniques	
		Direct	Indirect
Evaluation without dentures	Initial	3.20 (0.9) Aa	3.00 (1.2) Aa
	Immediately after relining	1.30 (0.6) Ba	1.90 (0.9) Ba
	30 d	2.10 (1.1) Ba	2.50 (0.5) Ba
	60 d	1.40 (0.6) Ba	2.60 (0.9) Ba
	90 d	1.80 (0.7) Ba	1.90 (0.6) Ba
	180 d	1.40 (0.7) Ca	1.50 (1.1) Ca
Evaluation with dentures	Initial	3.20 (0.9) Aa	3.00 (1.2) Aa
	Immediately after relining	1.30 (0.6) Ba	1.90 (0.9) Ba
	30 d	2.10 (1.1) Ba	2.50 (0.5) Ba
	60 d	1.40 (0.6) Ba	2.60 (0.9) Ba
	90 d	1.80 (0.7) Ba	1.90 (0.6) Ba
	180 d	1.40 (0.7) Ba	1.50 (1.1) Ba
Immediate evaluation without dentures after cleaning	Initial	1.90 (0.9) Aa	2.10 (1.5) Aa
	Immediately after relining	1.00 (0.7) Ba	0.80 (1.0) Ba
	30 d	0.70 (0.7) Ba	1.50 (0.7) Ba
	60 d	0.70 (1.8) Ba	1.40 (0.8) Ba
	90 d	1.10 (0.9) ABa	1.40 (0.7) ABa
	180 d	0.70 (0.5) Ba	1.20 (0.9) Ba
Immediate evaluation with dentures after cleaning	Initial	1.90 (0.9) Aa	2.30 (1.3) Aa
	Immediately after relining	1.00 (0.7) Ba	1.00 (1.0) Ba
	30 d	0.80 (0.6) Ba	1.50 (0.7) Ba
	60 d	0.70 (0.8) Ba	1.50 (0.8) Ba
	90 d	1.40 (0.8) ABa	1.50 (0.7) ABa
	180 d	1.80 (1.1) ABa	1.20 (0.9) Ba
Note: Tukey test ( *p* < 0.05). Different capital letters in vertical (column) show a statistically significant difference (in each assessment individually) ( *p* < 0.05). Different lower case letters in horizontal (line) show a statistically significant difference ( *p* < 0.05). Mean values < 3 were considered clinically acceptable. Mean values ≥3 were considered clinically unacceptable.			

## Discussion

According to Souza et al, OHIP-EDENT can be considered a good indicator of oral health-related quality of life for edentulous subjects. 25 It is worth mentioning that the OHIP-EDENT used in this study was validated for the Portuguese language (Brazil). 25

In the present study, there was no difference between the two relining techniques in all the evaluations, probably due to the fact that the relining material used was of long duration (up to 2 years). Therefore, as the patients were evaluated in a maximum period of 180 days, the material “durability” factor could have influenced the absence of difference between the techniques.

Despite the absence of difference between the techniques, the direct technique could be considered more advantageous by the dentist and patient, since it is simpler, quicker, 16 and avoids the laboratorial phase in which the patient remains without his or her complete denture. 11

As to the OHIP-EDENT questionnaire, in the questions related principally to the sensation of pain and function, in which the responses were initially “always” or “sometimes,” there was a change to “never” after 30 days of relining ( Table 1 ). Probably, the greater stability, retention, adaptation, and softness after relining could have contributed to a greater comfort, 2 5 7 9 16 a better biomechanical response, 9 a greater masticatory performance, 10 and a greater number of occlusal contacts 10 with more milling of food, which consequently could have helped in digestion, which according to Boland 2016 is a process that begins in the mouth. 26 Another important observation is that after relining, the uniform distribution of the masticatory stress on the alveolar ridge could have facilitated the maximum intercuspation without overloading a determined muscle more than another. 2 Thus, the quality of life increased after 30 days of relining ( *p* < 0.05) due to these probable factors. It is worth mentioning that between the time points 30, 60, and 180 days after relining, the quality of life was maintained, that is, there was no statistically significant change in quality of life between these time points ( *p* >0.05; Table 1 ).

As to the questionnaire of satisfaction with the relining, the satisfaction increased significantly after 30 days of relining with both techniques ( Table 2 ). It was observed that the category related to pain had the greatest influence on the results after 30 days of relining (pain reduction) ( *p* < 0.05; Table 2 ). This reduction in pain could have occurred due to better distribution of masticatory stresses on the ridge 2 5 7 10 and because the softness of the material (UFI Gel SC, VOCO, Germany) compensates the thickness and viscoelasticity lost from the mucosa overlying the bone. 10 It is important to remember that viscoelasticity and thickness of the mucosa are lost due to the resorption of the ridge with time. 10 Satisfaction was maintained between time points 30, 60, and 90 days after the relining for both techniques ( *p* >0.05; Table 2 ). After 180 days of relining, there was a reduction ( *p* < 0.05) in relation to satisfaction compared with time points 30, 60, and 90 days after relining **(** Table 2 ). There was no difference between the initial time points and 180 days after relining; however, after 180 days of relining, there was no return of painful sensation compared with the initial time point. The score for the satisfaction questionnaire with the relining in the final time point (after 180 days) was negatively influenced by reliner material in both techniques, in a way that there was a perception that parts of the relining material had come loose from the base of the inferior denture, probably due to the lack of chemical union between these materials. 2 20 27 [Bibr JR_27] 28

In the last two time points (90 and 180 days) of the evaluation, the occlusal force of the patients significantly greater when compared with the initial time point, for both techniques, and for the three regions evaluated ( *p* < 0.05; Table 3 ). This probably occurred since the effort, capacity, and the muscular force increased due to the inferior complete denture having good retention and stability after the relining. 2 A uniform distribution of occlusal force on the mucosa by the relining could have increased the capacity to support tensions of the mucosa, generating this significant increase in occlusal force. 10 In addition, the greater use times of the inferior complete denture with the reliner (90 and 180 days) could have generated an increase in adaptation, muscular capacity, and neuromuscular abilities of the patients, 17 generating this significant increase in occlusal force.

Halitosis, also known as oral malodor or bad breath, is characterized by unpleasant odor originating from the mouth of an individual and is noticed by others. 29 30 For evaluation without prior hygiene ( Table 4 ), immediately after relining (evaluation with and without denture), there was a significant reduction in halitosis when compared with the initial time point, for both techniques ( Table 4 ). In this reduction, the level of halitosis went from a clinically unacceptable situation (≥ 3) to a clinically acceptable situation (< 3). Possibly both relining techniques have contributed to a reduction in halitosis, since for the execution of both, a hygienization is performed during the process (hygiene is part of the techniques), as described in the methodology. It is worth remembering that due to the wear of the base of the inferior denture for the addition of the relining material, this leads to the removal of the microorganisms related to bad breath ^15,29^ (e.g., *Candida albicans* ). ^15,31,32,33^ Despite this, more studies are needed with different methodologies to confirm this result.

In evaluations with and without dentures (without prior hygiene), the initial halitosis level was clinically unacceptable in all situations (≥ 3; Table 4 ). This was different initially after cleaning, as in evaluations with and without dentures, halitosis became clinically acceptable in all situations (< 3; Table 4 ). This shows the importance of cleaning the oral tissue and dentures.

Another important point to be considered is that, for evaluations without prior hygiene of the mouth and dentures after 30, 60, 90, and 180 days of relining, the levels of halitosis remained clinically acceptable in all situations (for both techniques) (< 3; Table 4 ). Perhaps with the improvement in the patients’ quality of life, they started to take better care of their oral health. In addition, according to the reliner manufacturer (UFI Gel SC, VOCO, Germany), after relining, the growth of bacteria and fungi is minimized on the surface of the new silicone-based denture base, due to its surface smoothness and hydrophobic characteristic.

## Conclusion

Independent of the relining technique used, there was an increase in the quality of life, satisfaction with relining, and occlusal force, as well as a reduction in the level of halitosis. Both techniques generated similar results and therefore can be options in clinical practice. 28 34
